# Erythropoietic protoporphyria: case reports for clinical and therapeutic hints

**DOI:** 10.1186/s13052-023-01544-2

**Published:** 2023-11-23

**Authors:** Cristina Tumminelli, Francesca Burlo, Serena Pastore, Giovanni Maria Severini, Irene Berti, Stefano Marchini, Davide Zanon, Eleonora De Martino, Alberto Tommasini

**Affiliations:** 1 Department of Medicine, Surgery, and Health Sciences, University of Trieste, Trieste, Italy; 2 Department of Pediatrics, Institute for Maternal and Child Health “IRCCS Burlo Garofolo”, Via dell’Istria, 65, Trieste, 34137 Italy; 3 Department of Medical Genetics, Institute for Maternal and Child Health, IRCCS Burlo Garofolo, Trieste, Italy; 4 Department of Surgical and Medical Sciences for Children and Adults, Internal Medicine Unit, University of Modena and Reggio Emilia, Via del Pozzo 71, Modena, 41124 Italy; 5 Pharmacy and Clinical Pharmacology Department, Institute for Maternal and Child Health - IRCCS “Burlo Garofolo”, Trieste, Italy

**Keywords:** Porphyria, Photosensitivity, Cimetidine, Children porphyria, Ferrochelatase gene

## Abstract

**Background:**

Erythropoietic protoporphyria is a rare disorder which represents an important health problem in children, causing painful photosensitivity. Little is known on the correlation between genetic profile and clinical manifestations. The standard of care for Erythropoietic protoporphyria is based on avoiding sun and using sun protections, but recent literature has suggested that cimetidine may have a role in improving sun sensitivity. Herein we report our case series describing the successful use of cimetidine and analyzing potential phenotype-genotype correlations.

**Case presentation:**

This case series describes five patients presented to our Rheumatology Service complaining sun sensitivity. Blood exams and genetic analysis were consistent with the diagnosis of erythropoietic protoporphyria. Four of 5 patients received cimetidine in addition to standard therapies and the effect of treatment was evaluated by Erythropoietic Protoporphyria - Quality of Life questionnaire.

**Conclusions:**

Erythropoietic protoporphyria usually manifests in early childhood after a short sun exposure. Skin manifestations are the main reason for investigations, although sometimes they can be more subtle, leading to a significant diagnostic delay. Skin diseases in children can have profound effects on their family and social relationships. A treatment with cimetidine appears to be an excellent therapeutic option in children with Erythropoietic protoporphyria.

## Background

**Fig. 1:**
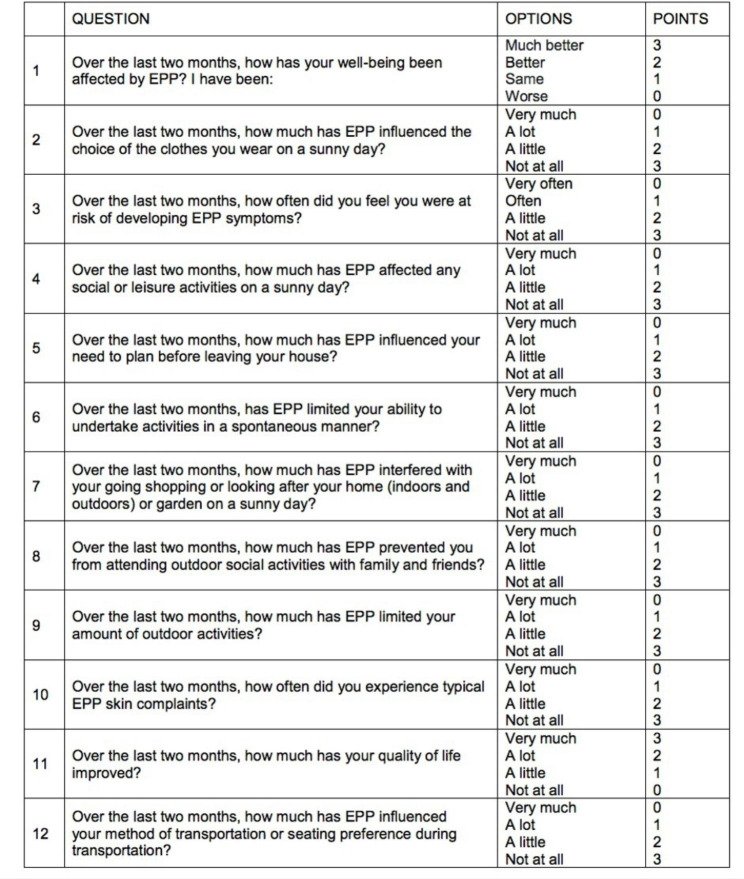
Erythropoietic Protoporphyria - Quality of Life (EPP-QOL) questionnaire. The questionnaire includes 12 items and 4 answer options. Depending on the answer there is a minimum (0) or maximum (3) score. The total score is between 0 and 36, higher scores indicating a better quality of life

Porphyria is a group of rare diseases caused by the altered biosynthesis of heme. Defects in the ferrochelatase (FECH), delta-aminolevulinate synthase 2 (ALAS2) or caseinolytic mitochondrial matrix peptidase chaperone subunit (CLPX) genes are associated with erythropoietic protoporphyria (EPP), a disease usually presenting in children, due to the accumulation of protoporphyrin-IX (PPIX) in erythrocytes, skin and liver [1]. Liver damage, which is the main risk in EPP, is rare in children, who usually complain only of symptoms related to sun exposure (burning, itching, pain), that can have significant impact on patients’ health-related quality of life (QoL) [2]. Treatment relies on the protection from sunlight and on the prevention of liver damage associated with protoporphyrin accumulation. We report a series of children cared for at the Rheumatology Service of IRCCS Burlo Garofolo Hospital in Trieste (Italy) describing clinical, biochemical, genetic features, and the effect of treatment with cimetidine on Erythropoietic Protoporphyria - Quality of Life (EPP-QOL) questionnaire (total score ranging 0 to 36, higher scores indicating better quality of life – Fig. 1) [3].

## Case presentation

Patient 1 is a 12-year-old girl with EPP diagnosed at the age of seven for a history of recurrent episodes of intense itching in the hands after sun exposure that did not improve after administration of antihistamines and associated with hyperemia and rarely edema of the dorsum of the hands and feet. On laboratory exams high erythrocyte protoporphyrins (14.55 µmol/L, normal value 4 µmol/L) and fecal protoporphyrins (217 nmol/g, normal < or = 151) were found. The diagnosis of EPP was confirmed by genetic analysis which found two heterozygous mutations of the FECH gene (c.67 + 5G > A, c.315-48T > C). The integration of carotenoids was suggested during the spring and summer period, and due to a transient increase in bile salts and a slight dilatation of the extrahepatic bile ducts, ursodeoxycholic acid was added to the therapy. However, liver magnetic resonance ruled out hepatic involvement of the disease. At the age of nine, the patient began a cyclic treatment with cimetidine during the spring and summer period, with a good clinical response on summer exposure tolerance.

Patient 2 is a 14-year-old girl with a ten-year history of photosensitivity, with a burning and tingling sensation on the skin within hours of exposure to sunlight. At the age of ten she developed a generalized erythematous and edematous skin reaction with petechiae on the backs of her hands and feet after a full day in the sun, unresponsive to oral antihistamine. After that episode she was evaluated at the Rheumatology Service where EPP was confirmed by the presence of elevated red cell protoporphyrins (13.2 µmol/l) and a heterozygous mutation on the FECH gene (c.315-48T > C). Since diagnosis, she has started hepatoprotective treatment with ursodeoxycholic acid, in addition to carotenoid supplementation. In addition, a cyclic treatment with cimetidine during the spring and summer period was started with improvements in sunlight tolerance.

Patient 3 is a 15-year-old boy with a seven-year history of suddenly occurring severe itching and pain in the hands and feet after sunlight exposure and physical exercise, lasting up to 48–72 h. No associated skin swelling, or redness were reported. At every episode, cetirizine was administered, without any improvements. Curiously, these episodes did not occur every time the boy was exposed to the sunlight. Allergic reaction and Fabry disease were ruled out and symptoms had been framed as erythromelalgia. Then, he was evaluated at the Rheumatology Service where EPP was diagnosed on the basis of high erythrocyte protoporphyrins (5.8 µmol/l) and two heterozygous variants of the FECH gene (c.757_761delAGAAG, c.315-48T > C). Then, hepatoprotective treatment with ursodeoxycholic acid was started, as well as carotenoids and vitamin D during the spring and summer period.

Patient 4 is 17 years old. When she was three, she started to present recurrent episodes of hyperemia, edema and itching on both hands and feet after sun exposure. When she was eleven, she was evaluated for the first time at the Rheumatology Service. She presented hepatomegaly, elevated liver enzymes, and high level of protoporphyrins (erythrocyte 35.69 µmol/l, fecal 1867 nmol/g). Genetic analysis confirmed the diagnosis of EPP, with two mutations on the FECH gene (c.400delA, c.315 − 48 T > C). At first, a treatment with ursodeoxycholic acid, vitamin D and carotenoids was started. Nonetheless, she always presented the same symptoms after sun exposure. Then, when she was 16, she started a cyclic treatment with cimetidine during the spring and summer period, with a consistent improvement of symptoms.

Patient 5 is a 13-year-old boy with a nine-year history of burning and itching on hands and feet after sun exposure. When he was twelve, he was evaluated at the Rheumatology Service for the first time, where EPP was diagnosed after the finding of high erythrocyte protoporphyrin (5.5 µmol/l) and either two splicing mutations (c.599-3 C > T and c.498 + 1 G > T) or a third variation (c.315-48T > C) on the FECH gene. Then, oral supplementation with carotenoids was suggested. When he was 13, he started a treatment with cimetidine during the spring and summer period, with a consequent better tolerance for sun exposure.

All patients treated with cimetidine (20 mg/kg/day in two doses) reported an improved tolerance to sunlight after cimetidine, with increased hours spent in outdoor activities. The EPP-QOL questionnaire was administered before and after two months from the beginning of treatment. Total scores of patients expressed as the percentage of the maximum (100%) quality are summarized in Table 1.

**Table 1:** EPP QoL questionnaire scores after the beginning of cimetidine

	score EPP QoL	
	Before cimetidine	After cimetidine
Patient 1	31%	69%
Patient 2	42%	89%
Patient 4	12%	78%
Patient 5	19%	80%

*Total score of EPP QoL questionnaire is 36 (equal to 100%). Total scores of questionnaires completed before and after the beginning of cimetidine are expressed as percentage in relation to the total score*

## Discussion and conclusions

In Italy, the prevalence of EPP is estimated of 3.15 cases per million persons, with an incidence of 0.13 cases per million persons / year [1]. EPP is characterized by a wide heterogeneity in age of onset, symptoms severity, and duration, leading to a significant diagnostic delay [4]. Rarely, there may be a complete absence of visible skin lesions (*patient 3 and 5)*. Although skin manifestations are the main reason for investigations, the most important complication in EPP is hepatic failure, due to the accumulation of protoporphyrin in the hepatobiliary structures. Approximately 1–5% of patients develop liver damage and require liver transplantation. However, this does not correct the underlying metabolic deficiency, and the transplanted liver is likely to have protoporphyrin damage [5]. In our cases, no patients developed consistent liver involvement in pediatric age. The diagnosis is based on clinical symptoms and increased levels of circulating protoporphyrin, while urinary porphyrins values ​​are usually normal [6]. In our cases, all patients had normal values of urinary porphyrins with increased erythrocyte levels and two of them increased fecal ones (*patient 1 and 4*). Genetic analysis is used to confirm the diagnosis of EPP. EPP can be caused by a compound heterozygous or homozygous mutation in FECH gene, but more often, it results from semi-dominant inheritance with compound heterozygous of two mutations, one of which is clearly pathogenic and the other having a minor contribute on the phenotype [7]. The genetic analysis performed on our patients agrees with the literature, although in one case (*patient 2*) only one heterozygous variant in the FECH gene was identified (c.315-48T > C), but, the possible presence of other variants in intronic regions or in the promoter have been hypothesized and therefore further investigations may be necessary. From this small case series, a correlation between genotype and clinical manifestations cannot be assumed. Nonetheless, Balwani et al. described a significant correlation between erythrocyte protoporphyrin levels and earlier age at onset, decreased sun tolerance, and increased risk of liver dysfunction [8]. Analyzing the characteristics of our patients and previous study on this topic, a direct correlation between blood protoporphyrin values ​​and skin phenotype was seen, regardless of the underlying genetic defect and the inheritance pattern. Although, in the study by Minder et al., liver injury was described as strongly associated with null FECH mutations and in patients with autosomal recessive protoporphyria, recent studies have shown that mutations in the FECH gene alone do not explain the severe liver disease phenotype, since the same mutations are present in asymptomatic family members [9].

Skin diseases in children can have profound effects on their QOL, interfering with family and social relationships. Currently, EPP therapy is based on reduction of sun exposure and the use of high protection factor creams in the spring-summer months. To prevent hepatopathy, it could be useful to promote biliary secretion with ursodeoxycholic acid and to interrupt the enterohepatic circulation reducing the circulating levels of protoporphyrin with cholestyramine. Other treatments gave contradictory results, such as transfusion of erythrocytes to suppress erythropoiesis and, hence, reduce the protoporphyrin level or plasmapheresis and extracorporeal albumin dialysis, for which there is a lack of studies documenting their efficacy.

Moreover, since protoporphyric hepatopathy may be triggered by another cause of liver disease, all of our patients were vaccinated against hepatitis A and B.

To improve sunlight tolerance, some treatment options are currently available, such as alfamelanotide and cimetidine. The safety and efficacy of alfamelanotide in children has not yet been established, while there are some published experiences on the use of cimetidine in children [10–12] (Table 2). Cimetidine inhibits ALAS, the first enzyme in the heme biosynthetic pathway, resulting in decreasing protoporphyrin levels. In our experience, four out of five patients underwent treatment with oral cimetidine, at a dose of 20 mg/kg/day divided in two daily administrations. Patient 3 did not start the treatment because he did not feel the need to increase sun exposure. PPIX levels were measured in two patients receiving cimetidine after three months of treatment. Curiously, these levels did not change; therefore, cimetidine showed a cutaneous but not a biochemical effect. None of the patients reported adverse effects with regards to clinical safety. They reported an increase in skin color, although this is not due to the drug but to the increased of sun exposure. No patients complained of photosensitivity deterioration.

**Table 2:** summary of the different papers which report the use of cimetidine in EPP in pediatric population

Author, Year	Daily dose/duration	Side effects reported	Sun tolerance during treatment	erythrocytes PpIX level	No patient/age	How was evaluated the photosensitivity
Heerfordt^10^, 2022	1200 mg/4 months	None	increased	41% decrease after 2 months 19% decrease after 4 months	1/ N.A.	questionnaire about (i) overall effect on photosensitivity (ii) time spent outside (iii) time to first photosensitivity symptoms (iiii)edema (iiiii) protective clothing
Kiberd,^11^ 2018	30 mg/kg / Not available	None	Increased	N.A.	1/ 4-year-old	Subjective increased tolerance
Tu, ^12^ 2016	30–40 mg/kg/ > 2 years	None	Increased	Data available for one patient in which 37% decrease after 1 year of treatment	3/ median age 9-year-old	Objective: improvement of actinic photodamage of the skin Subjective: - increased of number of hours of outdoor activities - Reduced the use of analgesic drugs

*N.A. not available*

The EPP-QOL questionnaires showed that the use of cimetidine led to an improvement in their QoL. However, a limitation of this study was the lack of a defined method of measuring the sun exposure time, except for activities of known duration (e.g., football matches or home-school path).

In conclusion, although its rarity, EPP should be excluded in all photosensitive children, especially when discomfort is disproportionate to the extent of the cutaneous lesions. Based on the literature and our clinical experience, a treatment with cimetidine is effective and safe in improving tolerance to sun exposure.

## Data Availability

The datasets used and/or analyzed during the current study are available from the corresponding author on reasonable request.

## List of abbreviations

FECH: Ferrochelatase
ALAS2: delta-aminolevulinate synthase 2
EPP: caseinolytic mitochondrial matrix peptidase chaperone subunit (CLPX erythropoietic protoporphyria
PPIX: protoporphyrin-IX
QoL: quality of life
EPP-QOL: Erythropoietic Protoporphyria-Quality of Life
