# Correction: Erythropoietic protoporphyria: case reports for clinical and therapeutic hints

**DOI:** 10.1186/s13052-024-01832-5

**Published:** 2024-12-15

**Authors:** Cristina Tumminelli, Francesca Burlo, Serena Pastore, Giovanni Maria Severini, Irene Berti, Stefano Marchini, Davide Zanon, Eleonora De Martino, Alberto Tommasini

**Affiliations:** 1 Department of Medicine, Surgery, and Health Sciences, University of Trieste, Trieste, Italy; 2 Department of Pediatrics, Institute for Maternal and Child Health “IRCCS Burlo Garofolo”, Via dell’Istria, 65, Trieste, 34137 Italy; 3 Department of Medical Genetics, Institute for Maternal and Child Health, IRCCS Burlo Garofolo, Trieste, Italy; 4 Department of Surgical and Medical Sciences for Children and Adults, Internal Medicine Unit, University of Modena and Reggio Emilia, Via del Pozzo 71, Modena, 41124 Italy; 5 Pharmacy and Clinical Pharmacology Department, Institute for Maternal and Child Health - IRCCS “Burlo Garofolo”, Trieste, Italy

**Italian Journal of Pediatrics (2023) 49:156**

10.1186/s13052-023-01544-2

The legend to Fig. 1 in the original article omitted to state that the Erythropoietic Protoporphyria - Quality of Life (EPP-QOL) questionnaire is reproduced with permission from the copyright holder CLINUVEL (clinuvel.com). The authors apologise for this omission.

